# Maternal experience of intimate partner violence and low birth weight of children: A hospital-based study in Bangladesh

**DOI:** 10.1371/journal.pone.0187138

**Published:** 2017-10-26

**Authors:** Jannatul Ferdos, Md. Mosfequr Rahman

**Affiliations:** Department of Population Science and Human Resource Development, University of Rajshahi, Rajshahi, Bangladesh; University of Bristol, UNITED KINGDOM

## Abstract

**Objective:**

Intimate partner violence (IPV) is the most prevalent form of gender-based violence worldwide. IPV either before or during pregnancy has been documented as a risk factor for the health of the mother and her unborn child. The aim of this study was to examine the relationship between maternal experience of IPV and low birth weight (LBW).

**Study design:**

A hospital-based survey was conducted among women in the postnatal wards of a large public hospital at Rajshahi, Bangladesh. Data on socio-economic characteristics, reproductive health characteristics, intimate partner violence, and antenatal, delivery and newborn care were collected from 400 women between July 2015 and April 2016.

**Results:**

Results of this study indicated that 43% of women reported experiencing any physical IPV in their lifetime, 35.5% of them experienced sexual IPV, and 32.5% experienced both physical and sexual IPV. Approximately one in every three (29.2%) infants was born with LBW. Physical IPV was associated with an increased risk of having a child with low birth weight (adjusted odds ratio [AOR]: 3.01, 95% CI: 2.35–5.81). The risk of infants born with LBW increased with women’s lifetime experience of sexual IPV (AOR: 1.98; 95% CI: 1.23–4.15) and both physical and sexual IPV (AOR: 4.05; 95% CI: 2.79–7.33).

**Conclusion:**

Maternal lifetime experience of IPV is positively associated with LBW children. Preventing women from the experience of IPV may help improve neonatal and child mortality in Bangladesh.

## Introduction

Low birth weight (LBW), defined by the World Health Organization (WHO) as birth weight of less than 2500 grams (5.5 pounds), is considered the most common risk factor for neonatal mortality [1]. A range of both short- and long-term consequences are associated with LBW. For instance, infants born with LBW are particularly more susceptible to frequent infections, malnutrition, poor cognitive development [2,3], higher risk of stunting by 2 years of age [4], leading to irreversible outcomes after age 3 years, including short height in adulthood, lowered immune function [5], an increased risk of chronic disease and reproductive complications in later life [6], as well as lower productivity in a range of educational and economic activities [6]. Moreover, using the developmental origins of health and disease (DOHaD) hypothesis, many studies have already shown that longer-term health risks associated with LBW include type 2 diabetes, hypertension, cardiovascular disease and obesity [7–9]. It is estimated that more than 20 million infants around the world, representing 16% of all births, are born with LBW [10]. Although the great majority of LBW births occur in low- and middle-income countries, and especially in the most vulnerable populations [11], it is now a global concern as some high-income countries are also faced with high rates for their context [12]. A recent regional estimate showed that the prevalence of LBW include 28% in South Asia, 13% in sub-Saharan Africa and 9% in Latin America. However, in Bangladesh the LBW prevalence is 22% [13].

Risk factors and causes of LBW are multifactorial, complicated, and vary among countries or regions. The main causes of low birth weight are pre-term delivery or intrauterine growth retardation (IUGR) or both [14]. Data from different regions of developing and developed countries show that in developing countries most LBWs are due to IUGR, whereas in developed and in African countries they are due to prematurity [15]. A recent estimate of the prevalence and number of LBW infants born who have either IUGR or are preterm in developing countries including Bangladesh found that more than two-thirds of LBW infants are due to IUGR and the remaining one-third is preterm [16]. Besides IUGR and preterm birth, some other factors are reported to be associated with LBW, such as maternal poor nutritional status, maternal anthropometric measures, anemia, birth spacing, socio-economic status, intimate partner violence and antenatal care in developing countries, especially in South Asia [14,17,18]. Understanding the factors affecting newborn’s health is crucial for developing effective public health programs and interventions to reduce neonatal and child mortality.

Intimate partner violence (IPV) is a serious, preventable public health problem that has a wide range of adverse consequences for women and the new born babies. In a multi-country study by Garcia-Moreno et. al. [19] reported that 15% to 71% ever-partnered women experienced physical or sexual IPV, or both, at some point in their lives. High rates of lifetime physical (42%-51%) and sexual (37%-50%) IPV against women in Bangladesh have been documented in several population-based studies [19,20]. The relationship between IPV and LBW has been documented in the earlier literature [21–26]. Some health-facility based studies in developing countries show noteworthy association between IPV and LBW but these studies considered physical/sexual IPV during pregnancy only and ignored lifetime experience of IPV [17,23–25]. A longitudinal cohort study in Bangladesh found that LBW was higher among women who reported different forms of family violence (by partner or other family members) [22]. However, this study was conducted only on rural women. The association between the experience of IPV and LBW infants was confirmed in several meta-analyses as well [27,28]. No association was also reported in some other studies. For example, in a case-control study, Grimstad et al. [29] found no association between physical IPV and LBW after adjusting for potential confounders. They suggested that stress could be a more powerful predictor of LBW than IPV. Their finding was supported by another study conducted in the U.S.A. [30], in which women who experienced IPV-related stress had odds of LBW twice as high, with mean birth weight 236 grams lower than women with no stress. Although among Afro-American low-income women, physical abuse was positively associated with LBW while sexual or emotional violence was not [31]. Research on the relationship between women’s experience of IPV and LBW, therefore, has been inconclusive which underscores the need for further investigation.

Several mechanisms, how the experience of IPV might lead to LBW, have been reported in previous studies. For example, experience of IPV involving abdominal trauma can lead to placental damage, preterm labor, rupture of the membranes, ruptured uterus, and placental abruption, all of which lead to preterm birth/LBW [17,32–34]. Women who experience IPV either during pregnancy or outside of pregnancy are found to be at higher risks of mood or anxiety disorder, depression, and posttraumatic stress disorder [35–37]. These factors have been linked to LBW [30,38,39]. Moreover, experience of IPV might lead to behavioral changes such as seeking less prenatal care/late entry into prenatal care, inadequate weight gain, smoking, drinking, and substance use during pregnancy, and these behavioral changes might result in LBW [17,34].

Studies on the relationship between IPV and low birth weight are limited in Bangladesh. Despite substantial progress in reducing child mortality, LBW remains one of the main causes of child mortality and morbidity [40]. The Sustainable Development Goals 3 (SDGs) aim to reduce neonatal mortality to at least as low as 12 per 1,000 live births, and under-5 mortality to at least as low as 25 per 1,000 live births by 2030 [41]. Bangladesh, therefore, needs to focus on all the factors that cause high neonatal and child mortality. In this study, we hypothesize that maternal lifetime experience of IPV increases the risks of LBW. We seek to investigate the association between maternal experience of IPV and LBW of the newborn using hospital-based data in Bangladesh.

## Methods

### Data

A hospital-based survey was conducted among women in the postnatal wards of Rajshahi Medical College Hospital, Rajshahi, Bangladesh. The rationale of choosing Rajshahi Medical College Hospital was that it is the largest public hospital in Rajshahi area, which provides primary to tertiary treatment to people of all socioeconomic strata. The survey was conducted from July 2015 to April 2016. With a 95% confidence interval and the prevalence (p) of IPV 58% [42], considering the precision 10% of the prevalence (p) and 10% of non-response rate, using the formula $n=\frac{Z_{\frac{\alpha }{2}}^{2}p(1-p)}{\varepsilon^{2}}$, the minimum required sample size was 309. But during the study period we enrolled 443 women in total. However, twenty-five women refused participating in this study; 11 women were not in a physical state to participate; interview of 7 women was not possible due to lack of confidentiality as their relatives refused to go away. Finally, data on background and socio-economic characteristics, reproductive health characteristics, intimate partner violence, and antenatal, delivery and newborn care were collected from 400 women (S3 File).

After obtaining permission from the authority of the hospital, the interviewer team proceeded to collect data. Participants were all recently delivered women (within three days post-delivery) irrespective of whether the delivery was normal vaginal or caesarean section. Since the main variable of interest of this study was LBW therefore decision was made to collect information after delivery. Moreover, data collection after delivery reduces the chance of missing cases. Women who were admitted for an abortion or with pregnancy complications and later discharged undelivered were excluded. Beds of the postnatal wards were numbered consecutively and women from those occupying beds were selected randomly. After obtaining written consent, the selected women were administered a pretested structured questionnaire by trained female interviewer. During this process, no relatives of the women were allowed to remain present and interviews were conducted confidentially in an empty room. This study was approved by the institutional review board of Rajshahi Medical College.

### Outcome

Birth weight of the newborn was our primary outcome of interest. A newborn whose birth weight was less than 2500 grams, regardless of their gestational age, was identified as low birth weight (LBW) child. Although clinically it is important to differentiate among low birth weight, premature, and small for gestational age (SGA); in resource-poor settings gestational age measurement is prone to inaccuracy as successful and quality routine ultrasound is rarely available [43]. Therefore, the current measurement of LBW continues to be a convenient surrogate for preterm and SGA births and a strong predictor of early mortality in resource-poor settings [44]. Exact gestational age was not possible to calculate since majority of the participants of this study had no early ultrasound records. Newborns were categorized according to their weight at birth: low birth weight (1: <2500g) or normal birth weight (0: ≥2500g).

### Predictor variables

Maternal lifetime experiences of different forms of IPV were the main predictor variables in this study. To measure lifetime IPV, data were collected from ever-married women on whether they had ever experienced one or more acts of physical or sexual violence, or both, by a current or former husband at any point of their lives. Perpetration of physical and sexual IPV was assessed via several questions. Prevalence and severity were measured by a modified Conflict Tactics Scale [45] that has been used extensively to measure spousal violence. Women who reported that their husbands engaged in any of the following behaviors at any time in their married life were classified as lifetime experience of physical IPV: (i) pushing, shaking, or throwing an object; (ii) slapping; (iii) pulling hair or twisting an arm; (iv) punching or hitting with a fist or something harmful; (v) kicking or dragging; (vi) choking or burning; or (vii) threatening or attacking with a knife or a gun. A positive answer to any of these questions indicated physical perpetration. The Chronbach’s α for this measure was 0.73. Perpetration of sexual IPV was indicated by a woman’s positive response to an item asking whether she had been physically forced to have sexual intercourse even when she did not want to. Three exposure variables were defined for this analysis: physical IPV versus no physical IPV, sexual IPV versus no sexual IPV, and physical and sexual IPV versus no physical and sexual IPV. However, women’s experience of physical or sexual IPV defined in this study was mutually non-exclusive.

### Confounding variables

Based upon the current literature on low birth weight and intimate partner violence we chose an a priori confounder set for analytic purpose of this study which include age, age at marriage, education, place of residence, working status, pregnancy intention, antenatal checkup, household decision-making autonomy, maternal BMI, and maternal height. These factors have all been associated with low birth weight [46–54] and experience of IPV [55–64] in previous research. Participant’s age was categorized as: <20 years, 20 to 24 years and 25–35 years (since none of the women in the sample was aged over 35 years). Age at marriage was categorized as married before 18 years of age and married at the age of 18 years or more. Educational level of respondents was defined in terms of the formal educational system of Bangladesh: primary education (1 to 5 years), secondary (6 years to 10 years) and higher (11 years or more). Participant’s place of residence was classified as urban or rural areas. Working status was classified as housewives, service or others (agricultural worker; poultry raising, cattle raising; home-based manufacturing etc.). Maternal pregnancy intendedness was determined by asking women to recall their feelings at the time of conception for the current birth. Women were asked whether the pregnancy had been wanted at that time, mistimed (i.e., it occurred earlier than desired) or unwanted (i.e., it occurred when no children, or no more children, were desired). Antenatal checkups were categorized as: less than 4 checkups and 4 or more checkups. Women’s household decision-making autonomy was based on responses to individual questions regarding who makes decisions in the respondent’s household about obtaining health care, large household purchases, visits to family or relatives, and child healthcare. The response options were as follows: (a) respondent alone, (b) husband alone (c) respondent and husband, (d) other person. For each question, a value of 1 was assigned if the response was (a) or (c), and 0 for (b) or (d). The values were then added, resulting in a score from 0 to 4 (Cronbach α = 0.89). Maternal BMI was classified as underweight (<18.5 kg/m^2^), normal (18.5–24.99 kg/m^2^) or overweight/obese (≥25.0 kg/m^2^). Height was a dichotomous variable indicating whether a woman was at least 145 cm tall or shorter than 145 cm. The cutoff point of below 145 cm is considered as short stature because most studies in developing countries have used this cutoff for screening high-risk women [64,65].

### Statistical analysis

Chi-square tests were used to investigate associations between birth weight and predictor and confounding variables. Association between maternal lifetime experience of IPV and low birth weight were estimated using logistic regression procedures. Logistic regression models were fitted to the data to model the crude associations between different forms of IPV and LBW. Confounding variables were then added into the models. This was done to observe how the addition of other confounding variables affected the relationship between different forms of IPV and LBW. Three fully multivariate logistic regression models were created to analyze the occurrence of LBW among women, with each model containing a different IPV predictor. All the confounding variables were entered into the multiple regression models simultaneously. Odds ratios (ORs) were estimated to assess the strength of the associations, and 95% confidence intervals (CIs) were used for significance testing. The multicollinearity of the variables was checked by examining the variance inflation factors (VIF), which was <2.0, indicating that multicollinearity was low. All statistical analyses were performed using Stata version 13.1/MP (StataCorp, LP, College Station, Texas, USA).

## Results

Our data indicate that a large number of women (58.7%) had secondary education, and 25.5% of women were engaged in some income generating works at the time of interview. One-fifth of the most recent births (20.5%) were unwanted and 10% were mistimed. Results also indicate that about one-third (29.2%) of the newborns’ weight at birth was less than 2500 grams. Forty-three percent women ever experienced physical IPV, 35.5% experienced sexual IPV, and 32.5% of women ever experienced both physical and sexual IPV from their husbands in their lifetime (Table 1). Table 1 also presents association of the selected variables with birth weight. Increasing maternal age, higher age at marriage, higher maternal education, higher number of children ever born, and higher participation in household decision makings was negatively associated with giving birth to a LBW infant. The rate of LBW children increased with decrease in women’s age. Women aged below 20 years were more likely to have a child with low birth weight than their older counterparts (aged 25 and over) (56.8% vs. 14.7%). Women who married before age 18 years experienced a high rate of LBW children than women who married at or over age 18 years (39.8% vs. 24.0%). Rate of LBW children was higher among women whose pregnancies were unwanted or mistimed than women whose pregnancies were wanted. Participation in household decision makings of women reduces the rate of experiencing LBW children than non-participation in household decision makings. Results also show that experience of IPV was positively associated with LBW. The proportions of low birth weight were higher among women who ever experienced physical IPV (56.4% vs. 8.8%), sexual IPV (64.8% vs. 9.7%), and both physical and sexual IPV (69.2% vs. 10.0%). Interestingly, the association between birth weight and some theoretically important variables such as education, children ever born, and maternal BMI showed no association between them (Table 1). This may be due to small sample size of this study.

**Table 1:** Association of selected variables with birth weight^a^.

Characteristics	Total sample (n = 400)	Birth weight		p (χ^2^ -Test)
		<2500 gm, % (n = 117)	≥2500 gm, % (n = 283)	
**Age**				<0.001
<20	74 (18.5)	42 (56.8)	32 (43.2)	
20–24	175 (43.9)	52 (29.7)	123 (70.3)	
25–35	150 (37.6)	22 (14.7)	128 (85.3)	
**Age at first marriage**				0.001
<18 years	133 (33.3)	53 (39.8)	80 (60.2)	
≥18 years	267 (66.7)	64 (24.0)	203 (76.0)	
**Place of residence**				0.085
Rural	251 (62.8)	81 (32.3)	170 (67.7)	
Urban	149 (37.2)	36 (24.2)	113 (75.8)	
**Respondents education**				0.134
Primary	64 (16.0)	25 (39.1)	39 (60.9)	
Secondary	235 (58.7)	67 (28.5)	168 (71.5)	
Higher	101 (25.3)	25 (24.8)	76 (75.2)	
**Respondents working status**				<0.001
Housewife	298 (74.5)	101 (33.9)	197 (66.1)	
Service	87 (21.7)	8 (9.2)	79 (90.8)	
Others	15 (3.8)	8 (53.3)	7 (46.7)	
**Total children ever born (CEB)**				0.215
1	234 (58.5)	74 (31.6)	160 (68.4)	
≥2	166 (41.5)	43 (25.9)	123 (74.1)	
**Pregnancy intention**				<0.001
Wanted	278 (69.5)	28 (10.1)	250 (89.9)	
Mistimed	40 (10.0)	21 (52.5)	19 (47.5)	
Unwanted	82 (20.5)	68 (82.9)	14 (17.1)	
**Participation in household decision makings**				<0.001
Not participated	131 (32.8)	58 (44.3)	73 (55.7)	
Participated at least one decision	269 (67.2)	59 (21.9)	210 (78.1)	
**Number of antenatal checkup**				0.036
<4	73 (18.3)	14 (19.2)	59 (80.8)	
≥4	327 (81.7)	103 (31.5)	224 (68.5)	
**Maternal BMI**				0.636
Normal weight	214 (53.5)	60 (28.0)	154 (72.0)	
Under weight	10 (2.5)	2 (20.0)	8 (80.0)	
Over weight	176 (44.0)	55 (31.2)	121 (68.8)	
**Maternal Height**				0.004
<145 cm	89 (22.2	37 (41.6)	52 (58.4)	
≥145 cm	311 (77.8)	80 (25.7)	231 (74.3)	
**Experience of physical IPV**				<0.001
Yes	172 (43.0)	97 (56.4)	75 (43.6)	
No	228 (57.0)	20 (8.8)	208 (91.2)	
**Experience of sexual IPV**				<0.001
Yes	142 (35.5)	92 (64.8)	50 (35.2)	
No	258 (64.5)	25 (9.7)	233 (90.3)	
**Experience of both physical and sexual IPV**				<0.001
Yes	130 (32.5)	90 (69.2)	40 (30.8)	
No	270 (67.5)	27 (10.0)	243 (90.0)	

^a^ Values are given as number (percentage) unless otherwise indicated.

Table 2 presents the unadjusted and adjusted associations between maternal lifetime experience of different forms of IPV and low birth weight. There was a positive relationship between low birth weight and all forms of IPV. In unadjusted analyses, women who ever experienced different forms of IPV were more likely to have a LBW child. In adjusted analyses, results also indicated that women experiencing physical IPV (AOR: 3.01, 95% CI: 2.35–5.81) or sexual IPV (AOR: 1.98; 95% CI: 1.23–4.15) were at increased risk of having a LBW child. Women experiencing both physical and sexual IPV were more likely to report to have a LBW child (AOR: 4.05; 95% CI: 2.79–7.33) than women who did not experience both physical and sexual IPV.

**Table 2:** Logistic regression analysis to predict the association between maternal lifetime experience of IPV and low birth weight (n = 400).

	Crude OR (95% CI)	Adjusted OR^a^ (95% CI)
Physical IPV	4.45 (1.79–7.45)	3.01 (2.35–5.81)
Sexual IPV	2.80 (1.01–5.90)	1.98 (1.23–4.15)
Both physical and sexual IPV	5.41 (2.90–7.46)	4.05 (2.79–7.33)

Abbreviations: OR, odds ratio; CI, confidence interval; IPV, intimate partner violence.

^a^ Adjusted for maternal age, age at marriage, place of residence, maternal education, occupation, pregnancy intention, participation in household decision-makings, number of antenatal checkup, maternal BMI and maternal height.

## Discussion

This study findings support our hypothesis that maternal lifetime experience of IPV increases the probability of giving birth to a child with low birth weight. In this study, we found that nearly one-third of babies were born with low weight and the lifetime experience of IPV among women was also high. Moreover, proportion of LBW children was high among women who experienced different forms of IPV in their lifetime. This high lifetime prevalence rate confirms that IPV is alarmingly commonplace in this impoverished South Asian nation, potentially affecting the health of both mothers and their unborn children. The study results show that women who ever experienced physical IPV were three times more likely to have children with low birth weight than women who were not physically abused. However, the risk of producing a low birth child was even higher when a woman experienced both physical and sexual IPV (4 times more likely than a woman who did not experience physical and sexual IPV). Therefore, this study urges to the government or policy makers to formulate appropriate policies and to implement them properly to prevent women from being abused by their husbands, which might ensure good health for women as well as their children.

Our findings are consistent with the findings of previous studies [21,23,27] indicating that women with a history of physical violence by their partners are at greater risk of having low birth weight infants. Maternal experience of physical IPV might have direct or indirect consequences on the fetus resulting in low birth weight babies. The experience of physical IPV may have direct adverse consequences, such as abdominal trauma and placental damage, premature rupture of membranes [66], or release of prostaglandin which is associated with preterm labor and LBW [67]. Maternal experience of physical IPV may affect birth weight indirectly in several ways. Stress could be a mediating factor of the relationship between IPV and LBW by activating neuroendocrine axis, causing the release of catecholamines, betaendorphic and cortisol which can lead to vasoconstriction, fetal hypoxia, and fetal growth restriction [68]. Moreover, women exposed to IPV experience depressive symptoms, suicidal ideation, anxiety, and high levels of stress [22], attend fewer prenatal care visits, make poor food choices, and isolate themselves [69] which in turn might produce a low birth weight infant. Another explanation for the relationship of LBW to IPV is that these births could be unintended [63] which lead women to lower use of antenatal care [70] and lower weight at birth [54]. However, physically abused women are at greater risk for poor obstetric history and short interpregnancy interval, poor prenatal weight gain, anemia, first- or second-trimester bleeding, and infections [71]. These factors could be the potential reasons of the relationship between IPV and low birth weight.

Our findings also indicate that women who were sexually abused were almost two times more likely to give birth to low birth weight babies than non-abused women. These findings are in contrast with earlier studies [31,72] which found no associations between sexual violence and low birth weight in the adjusted analysis. The lack of a validated instrument for measuring the exposure, lower disclosure rate, lack of information on the context and frequency of the violence or information regarding the perpetrator could be possible explanations for lack of associations found in those studies. Studies that have investigated the impact of lifetime experience of sexual violence on low birth weight specifically are limited. However, results from a new meta-analysis published in the recent WHO report [73] have demonstrated a positive association between lifetime experience of physical and/or sexual violence and low birth weight. Our study reaffirmed that LBW may also stem from the fact that women exposed to sexual IPV experience immediate complications such as bleeding and rupture of membranes [62], which can lead to a preterm birth and LBW infant as well. Moreover, women might develop psychological disorders such as depressive symptoms, post-traumatic stress disorder or anxiety due to the experience of sexual violence from their husbands at any point of their lives [35,36], which in turn result in LBW children [38,39].

The results of our study should be interpreted in the light of several limitations. This study was carried out in one hospital only, and therefore, we cannot generalize the results to the whole country. Women were asked to self-report the experience of violence from their husbands; therefore, there could be a possibility of under reporting of IPV. Underreporting of IPV might also be due to the sensitive nature of the topic, societal acceptance, social stigma, and participants’ privacy and safely concerns. Another limitation is that the definition of LBW used in this study fails to distinguish between LBW infants who are premature and those who are merely small for their gestational age. IUGR, the predominant cause of LBW in Bangladesh, was not assessed in this study. Another important limitation of this study was that data was collected after delivery which may reduce the number of missing cases but it might introduce a recall and selection bias. Given the retrospective nature of the data, there is a possibility that women are biased towards recalling their experience of lifetime IPV. Moreover, it is evident that memory for events, even violent ones, fades over time [74], and therefore retrospective instrument used in this study is subject to substantial recall biases. Additionally the potential for selection bias in this study is that the study focuses on only women who delivered in a hospital whereas majority of the deliveries (62%) take place in home in Bangladesh [75]. Finally, since inferring causation is difficult due to the observational nature of the data, this study provides evidence of statistical association between the items of interest and the experience of IPV. Future in depth longitudinal research is needed to determine the magnitude of the relationship between IPV and LBW infants, and the mechanisms through which IPV leads to LBW.

Despite these limitations, the study findings have important policy implications and far-reaching consequences for child healthcare in Bangladesh. Since maternal experience of IPV is associated with LBW, therefore, we suggest policy makers to address the maternal experience of IPV while formulating policies towards reducing infant and child mortality and morbidity. The results of this study may also be relevant to other resource-poor countries where infant and child mortality rates are high, and to clinicians who assess newborns with health problems. We recommend additional efforts are needed from both the governmental and nongovernmental organizations to prevent IPV and promote reproductive health programs for them. Proper screening of IPV is needed to provide counseling and other social support to women in crisis. In this regard, gynecological and obstetric services may be key intervention points to screen IPV.

### Conclusions

To our knowledge, this study is the first hospital-based study in Bangladesh to document that maternal experience of IPV is positively associated with producing a low birth weight child. These findings emphasize the importance of screening all women of reproductive age. Bangladesh is striving towards SDGs 3 of reducing global neonatal and under five mortality. Therefore this study makes an important contribution by documenting that maternal experience of IPV could be a substantial impediment in achieving the SDG. IPV related child morbidity and mortality is a critically important health issue, particularly in South Asia, where neonatal mortality is relatively high. Therefore, global concerted initiatives should be taken and properly implemented to protect women from spousal violence and reduce child morbidity and mortality.

## Supporting information

S1 File Interview questionnaire in Bangla. (DOC)

S2 File Interview questionnaire in English. (DOC)

S3 File Complete dataset. (XLSX)

S4 File Label for variables in the dataset. (XLSX)
