# Supplementary material for: Maternal experience of intimate partner violence and low birth weight of children: A hospital-based study in Bangladesh
Source: PLoS One. 2017 Oct 26;12(10):e0187138. doi: 10.1371/journal.pone.0187138 (PMC5658163; doi:10.1371/journal.pone.0187138)
Supplement: S1 File — (DOC) [file pone.0187138.s001.doc]

***ccy‡jkbmv‡qÝ GÛ wnDg¨vbwi‡mvm© †W‡fjc‡g›U wefvM, ivRkvnx wek¦we`¨vjq***


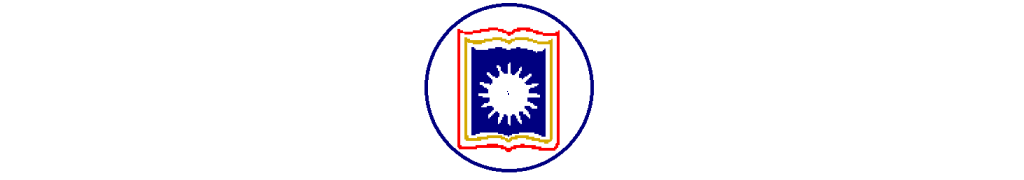


**M‡elYvi wk‡ivbvg: Impact of Intimate Partner Violence During Pregnancy on Delivery**

**Complications and Birth Outcomes: Study of Hospital Based Data in Bangladesh.**

**M‡elYvi cªkœgvjv :**

µwgK bs- ..........................আইডি.........................ZvwiL :............................mgq :...............................

**Av_©-mvgvwRK msµvšÍ Z_¨vejx**

1. evm¯nvb: K) MÖvg L) kni

2. eZ©gvb eqm ( c~Y© ermi) :...............eQi

3. ˆeevwnK Ae¯_v:

K) weevwnZ L) AweevwnZ M) weevn we‡”Q` N) weaev O) Ab¨vb¨

4. weev‡ni mgq eqm:..................eQi

5. Avcwb KZ ermi covïbv K‡i‡Qb ?............eQi

6. Avcbvi ¯^vgx KZ ermi covïbv K‡i‡Qb ?..............eQi

7. ag©: K) Bmjvg L) wn›`y M) Ab¨vb¨

8. Avcbvi mšÍvb KZ Rb : †gvU............

RxweZ............Rb

9. DËi`vZvi †ckv:

ক) বাড়ীর কাজ খ) চাকুরি গ) অন্যান্য (উল্লেখ করুন)............।

10. DËi`vZvi ¯^vgxi †ckv:

K) K…wl L) PvKzix M) kªwgK N) e¨emv O) Ab¨vb¨ (উল্লেখ করুন) ...........

11. M„nv¯’vwji cÖ‡qvRbxq wRwbm µq weµ‡qi wm×všÍ Kvi KvQ †_‡K wb‡q _v‡Kb?

K) wb‡R L) ¯^vgxi M) wb‡R I ¯^vgxi mv‡_ Av‡jvPbv K‡i N) Ab¨vb¨

12.AvZ¥xq ¯^R‡bi evwo‡Z †eov‡Z hvIqvi wm×všÍ Kvi KvQ †_‡K wb‡q _v‡Kb ? জানি ন

K) wb‡R L) ¯^vgxi M) wb‡R I ¯^vgxi mv‡_ Av‡jvPbv K‡i N) Ab¨vb¨

13.cwievi cwiKíbv I ¯^v¯n¨ msµvšÍ wm×všÍ Kvi KvQ †_‡K wb‡q _v‡Kb?

K) wb‡R L) ¯^vgxi M) wb‡R I ¯^vgxi mv‡_ Av‡jvPbv K‡i N) Ab¨vb¨

14.Avcbvi mšÍv‡bi ¯^v¯n¨ wm×všÍ Kvi KvQ †_‡K wb‡q _v‡Kb?

K) wb‡R L) ¯^vgxi M) wb‡R I ¯^vgxi mv‡_ Av‡jvPbv K‡i N) Ab¨vb¨

**Mf©Kvjxb ¯^v¯’¨ msµvšÍ Z_¨vejx**

15. Avcwb KZ eQi eq‡m 1g Mf©aviY K‡i‡Qb ?......................eQi|

16. me©‡kl mšÍvb Mf©ve¯’vq Avcwb †gwW‡Kj †PKAvc K‡i‡Qb wK ? n¨uv / bv

n¨uv n‡j KZ evi ..........

17. gvZ…Z¡Kvjxb mg‡q Avcwb †Kv_vq wPwKrmv wb‡q‡Qb?

K) nvmcvZvj L) wK¬wbK M) evwo N) ¯^v¯’¨‡K›`ª

18.cÖ_g †PKAvc mg‡q Avcbvi IRb KZ wQj ?..................... ‡KwR........................ জানিনা

19. mšÍvb cÖm‡ei c~‡e© IRb KZ wQj ?................................ ‡KwR........................ জানিনা

20.Avcbvi D”PZv KZ wQj?.............................. সে. মি....................... জানিনা

21.me©‡kl mšÍvb Mf©ve¯’vq Avcwb Blood pressure †PKAvc K‡i‡Qb wK ? n¨uv / bv

DËi n¨uv n‡j Wv³vi / bvm© Avcbv‡K e‡j‡Q Avcbvi D”Pi³Pvc Av‡Q? n¨uv / bv

22. me©‡kl mšÍvb Mf©ve¯’vq Avcbvi i³Pvc KZ wQj? (মেডিক্যাল রেকর্ড চেক করুন)

K) Systolic blood pressure………………… mmHg

L) Diastolic blood pressure……………… mmHg

23.me©‡kl mšÍvb Mf©Kvjxb c~‡e© Avcbvi Wvqv‡ewUm wQj wK ? n¨uv / bv

24.Wv³vi / bvm© wK Avcbv‡K me©‡kl mšÍvb Mf©Kvjxb Wvqv‡ewUm Av‡Q e‡j‡Qb wK? n¨uv / bv

me©‡kl †PKAv‡ci mgq (মেডিক্যাল রেকর্ড চেক করুন) Random Plasma Glucose (RBS)………………mmol;

Fasting Blood Sugar……. …… …………..mmol

25.me©‡kl mšÍvb Mf©Kvjxbmg‡q wK †Kvb mgm¨v wQj? n¨uv / bv

DËi n¨uv n‡j

K) nvZ cv R¡vjvKiv L) kixi PzjKvbI M) i³ ¯^íZv N) cÖmv‡e mgm¨v O) nvZ cv dz‡j hvIqv P) †ncvUvBwUm ( RwÛm ) Q) Ab¨vb¨ (উল্লেখ করুন) ...............

26.me©†kl mšÍvb cÖm‡ei mg‡q wK †Kvb mgm¨vq c‡o‡Qb wK? n¨uv / bvÔ

n¨uv n‡j, †Kvb ai‡bi (উল্লেখ করুন, মেডিক্যাল রেকর্ড চেক করুন)...........................|

**R‡b¥i c‡i wkïi hZœ mµvšÍ Z_¨vejx**

2৭. আপনার সর্বশেষ সন্তান গর্ভধারণ এর সময়, আপনি কি সে সময় গর্ভধারণ করতে চেয়েছিলেন, পরে হতে চেয়েছিলেন, কখনই হতে চাননি?

K ) †mB mgq B”Qv wQj L) cieZx©‡Z B”Qv wQj M) Avi B”Qv wQj bv|

2৮. me©‡kl mšÍvb‡K KZ gvm M‡f©aviY K‡iwQ‡jb ?.................. gvm |....................... সপ্তাহ

২৯. me©†kl mšÍvb cÖm‡ei c‡i mšÍvb IRb KZ wQj ? (মেডিক্যাল রেকর্ড চেক করুন).....................‡KwR|

৩০. R‡b¥i c‡i Avcbvi mšÍv‡bi †Kvb mgm¨v / RwUjZvq c‡o‡Qb wK ? n¨uv / bv

৩১. n¨uv n‡j, কোন ধরনের জটিলতা (উল্লেখ করুন, মেডিক্যাল রেকর্ড চেক করুন)………………………………………

**স্বামী কর্তিক নির্যাতন**

৩২. Avcwb wK কখনও আপনার বর্তমান অথবা পূর্বের স্বামীর Øviv নিম্নের কোন নির্যাতন এর wkKvi n‡q‡Qb ?

|  | **Rxe‡b †h ‡Kvb mg‡qi** | | **me©†kl Mf©Kvjxb mg‡q** | |
| --- | --- | --- | --- | --- |
|  | **n¨uv** | **bv** | **n¨uv** | **bv** |
| av°v / SwK, ‡Kvb wKQ zQz‡o gviv? |  |  |  |  |
| Po gviv? |  |  |  |  |
| nvZ gyP‡o †`qv / Pzj a‡i Uvbv? |  |  |  |  |
| wKj gviv / Nywl gviv? |  |  |  |  |
| jvw_ gviv/m‡Rv‡i Uvbv ev gviv? |  |  |  |  |
| ‡Kvb Kvi‡Y cywo‡q †`qv / k¦vm‡iva Kiv? |  |  |  |  |
| Qzwi ,wc¯Íj A_ev †Kvb aviv‡jv A¯ ¿ w`‡q fq †`Lv‡bv? |  |  |  |  |
| Avcbvi B”Qvi weiƒ‡× ‡hŠbwgj‡bi জন্য kvwiwiK Rei দস্তির wkKvi n‡q‡Qb wK? |  |  |  |  |

**Avcbvi gZvg‡Zi Rb¨ ab¨ev` !**
