# Supplementary material for: Maternal experience of intimate partner violence and low birth weight of children: A hospital-based study in Bangladesh
Source: PLoS One. 2017 Oct 26;12(10):e0187138. doi: 10.1371/journal.pone.0187138 (PMC5658163; doi:10.1371/journal.pone.0187138)
Supplement: S2 File — (DOC) [file pone.0187138.s002.doc]

Department of Population Science and Human Resource Development, University of Rajshahi


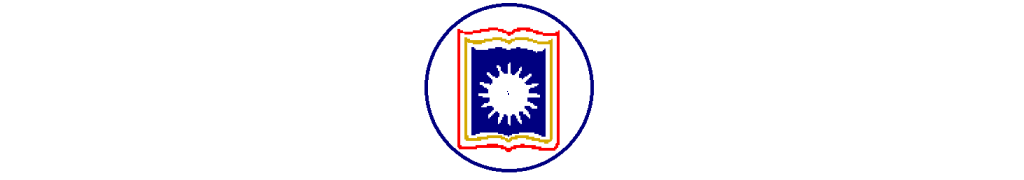


Research Title: **Impact of Intimate Partner Violence on Delivery Complications and Birth Outcomes: Study of Hospital Based Data in Bangladesh**

**QUESTIONNAIRE**

Serial No .................ID No.......................Date:............................Time:………………...

**Socio-Economic Characteristics**

1. Place of residence: a) Rural b) Urban

2. How old were you at your last birthday (Age in completed years)?: ……….Years

3. What is current mariatal status?

a) currently married b) Separated c) Divorced d) Widowed

4. How old were you at your first marriage (completed years)? …………………Years

5. What is the highest year of education (completed years)?.....................years

6. What level of schooling did your husband last attended (completed years)?..............years

7. What is your religion?

a) Islam b) Hindu c) Others

8. How many children have you given birth including the present one who were alive when they are born?.......

How many of them still alive?....................

9. What is your occupation, that is the kind of work do you mainly do?

a) Household work b) Service c) Others (please specify)….

10. What is your husband’s occupation, that is what kind of work does he mainly do?

a) Agricultural work b) Service c) Laborer d) Business e) Others (please specify)………

11. Who usually makes decisions about making major household purchases?

a) Respondent b) Husband c) Respondent and husband jointly d) Others

12. Who usually makes decisions about visits to your fmily or relaives?

a) Respondent b) Husband c) Respondent and husband jointly d) Others

13. Who usually makes decisions about health care for yourself?

a) Respondent b) Husband c) Respondent and husband jointly d) Others

14. Who usually makes decisions about your child health care?

a) Respondent b) Husband c) Respondent and husband jointly d) Others

**Reproductive Health**

15. How old were you when you were pregnant for the first time? Age in completed years………

16. For last pregnancy, have you ever visited health center/doctors/clinics for antennal check up?

a) Yes b) No

If yes, how many times?.............

17. Where did you take antenatal care?

a) Govt. hospital b) Clinic c) Community health centers d) Other places

18. How much did you weigh at first ANC visit?.....................KG……………..Don’t know.

19. How much did you weigh before birth?..................................KG………………….Don’t know

20. What is your height?.......................cm………………..Don’t know

21. Did you check blood pressure during last pregnancy? a) Yes……. b) No…….

If Yes, did doctor/nurse tell you that you have high blood pressure? a) Yes………..b) No……….

22. In last checkup, how was your blood pressure (Please check medical record)?

a) Systolic blood pressure……….mmHg;

b) Diastolic blood pressure….…..mmHg

23. Did you have diabeter before pregnancy? a) Yes…………..b) No……………

24. During last pregnancy, did doctor/nurse tell you that you have gestational diabetes?

a) Yes…………….b) No…………………..

If yes (Please check medicl record), a) Radom blood suger…………mmol;

b) Fasting blood suger…………mmol.

25. Did you face any problem during last pregnancy? a) Yes…………b) No………..

If yes, is the specific problems are:

a) Inflammation b) Allergy c) Anemeia d) Urinary problem e) Edema f) Jaundice h) Others (please specify)……….

26. Did you have any complications during last delivery? A)Yes…………b) No……..

If yes, (Specify, Please check medical records)……………

**Information of Last Birth**

27. For last birth, at the time you became pregnant, did you want to become pregnant then, did you want to wait until later, or did you not want to have any (more) children at all?

a) Then b) Later c) Not at all.

28. How many months or weeks along was the pregnancy?

.................Months/………………..Weeks

29. What was the birth weight of the baby (Please check medical record)?......................Kg

30. Did the baby have any malformation? a)Yes…………b)No………..

31. What kind of malformation did the baby have (Please specify, please check medical records)?.....................

**Intimate Partner Violence**

32. (Does/did) your current/former husband ever do any of the following things to you:

|  | **Ever** | | **During last pregnancy** | |
| --- | --- | --- | --- | --- |
|  | **Yes** | **No** | **Yes** | **No** |
| Push you, shake you, or throw something at you? |  |  |  |  |
| Twist your arm or pull you hair? |  |  |  |  |
| Slap you? |  |  |  |  |
| Punch you with his fist or with something that could hurt you? |  |  |  |  |
| Kick you, drag you or beat you up? |  |  |  |  |
| Try to choke you or burn you on purpose? |  |  |  |  |
| Threaten or attacj you with knife, gun, or any other weapon? |  |  |  |  |
| Physically force you to hve sexual intercourse with him even when you did not want to? |  |  |  |  |

**Thank you for your valuable time!**
